# Clinical Outcome of Carbon Fiber Reinforced Polyetheretherketone Plates in Patients with Proximal Humeral Fracture: One-Year Follow-Up

**DOI:** 10.3390/jcm12216881

**Published:** 2023-10-31

**Authors:** Patrick Ziegler, Sven Maier, Fabian Stuby, Tina Histing, Christoph Ihle, Ulrich Stöckle, Markus Gühring

**Affiliations:** 1 Department of Trauma and Reconstructive Surgery, Klinik Gut, 7500 St. Moritz, Switzerland; 2 Department of Trauma and Reconstructive Surgery, BG Unfallklinik Tuebingen, Eberhard Karls University Tuebingen, Schnarrenbergstrasse 95, 72076 Tuebingen, Germany; 3 BG Trauma Center, Department for Traumatology, Orthopedics and Surgery, 82418 Murnau am Staffelsee, Germany; fabian.stuby@bgu-murnau.de; 4 Center for Musculoskeletal Surgery, Charité University Medicine Berlin, 10117 Berlin, Germany; 5 Kronprinzenbau Klinik, 72764 Reutlingen, Germany

**Keywords:** proximal humeral fracture, PEEK, complications, postoperative outcomes

## Abstract

Background: Proximal humerus fractures are seen frequently, particularly in older patients. The development of new osteosynthesis materials is being driven by the high complication rates following surgical treatment of proximal humerus fractures. Plate osteosyntheses made of steel, titanium and, for several years now, carbon fiber-reinforced polyetheretherketone (CFR-PEEK) are used most frequently. Methods: A prospective, randomized study was conducted in order to evaluate whether there are differences in the functional postoperative outcome when comparing CFR-PEEK and titanium implants for surgical treatment of proximal humerus fractures. The primary outcome of shoulder functionality 1 year after surgery was measured with the DASH score, the Oxford Shoulder Score, and the Simple Shoulder Test. Results: Bony consolidation of the respective fracture was confirmed in all the patients included in the study within the scope of postoperative follow-up care. No significant differences in the DASH score, Oxford Shoulder Score, or Simple Shoulder Test were observed 1 year post-operatively when comparing the implant materials CFR-PEEK and titanium. Conclusions: There are no differences in terms of the functional outcome between CFR-PEEK plates and titanium implants 1 year after surgery. Studies on the long-term outcomes using CFR-PEEK plates in osteoporotic bone should be the subject of further research.

## 1. Introduction

Injuries to the shoulder are of great importance due to their high incidence and the heterogeneous patient population. Demographic changes with an aging society and a rising incidence of sports injuries are of importance. Proximal humerus fractures represent a common injury in humans and make up 4–5% of all fractures and up to 15% of fractures in patients over 65 years of age [1,2,3,4]. Despite numerous advances in surgical technology and innovations in the field of implants and osteosynthesis materials used in the last few decades, complication rates of up to 49% demonstrate the need for continuous improvement and further development of surgery–orthopedic care of proximal humerus fractures [5,6,7,8].

The goal when treating patients with proximal humerus fractures is complete restoration or improvement in musculoskeletal system functionality and attainment of an adequate quality of life. Various conservative and surgical treatments are available. In the context of surgical and head preservation treatment of proximal humerus fractures in adults, locking plate osteosynthesis and intramedullary nailing are the most common techniques. The introduction of locking implants and the resulting increased osteosynthesis stability improved results. Treatment with plates and open reduction and locking plate osteosynthesis became the standard surgical treatment for proximal humerus fractures [9,10,11,12,13]. With regards to materials selection and osteosynthesis properties, as well as surgical techniques, these procedures are subject to constant change with the aim of making treatment easier and improving the postoperative outcome. Frequent use of plate osteosyntheses historically showed high complication rates in the postoperative follow-up period [14,15,16]. Studies indicate that plate osteosynthesis can lead to complications requiring revision, e.g., secondary tilting of the fracture with subsequent screw penetration through the head (17%) [17,18,19]. These complications are especially prevalent in an elderly population with poor bone quality.

Plates made from carbon fiber-reinforced polyetheretherketone (CFR-PEEK) have been on the market for some years. The benefits of this thermoplastic material are radiolucency, no cold welding at the titanium screw–plate interface, and greater elasticity with the aim of increased micro-motion in the fracture gap. Although fewer secondary varus dislocations are described by Schliemann et al., the studies published to date do not show improved postoperative functional outcomes when using plates made of CFR-PEEK compared to titanium plates [20,21,22,23]. While increased elasticity compared to the titanium plate was confirmed in biomechanical studies, the question as to whether this elasticity offers an advantage in all fracture types is currently the source of much debate [23,24].

The aim of this study was to compare the postoperative outcome of patients with a proximal humerus fracture treated with a locking plate made from CRF-PEEK or titanium.

## 2. Materials and Methods

The study was registered at the German Register of Clinical Trials in Freiburg (DRKS00011376) and the protocol was approved by the local ethics committee (347/2016MP1). All patients included in this study gave consent to participation in writing.

Between October 2016 and June 2018, 76 patients treated for proximal humerus fractures at the BG Hospital Tübingen were included in the study and randomized to the titanium group or the CFR-PEEK group by means of a randomization list. There was no blinding of the patients, surgeons, or investigators.

The randomization list was generated before the start of the study using the “random number” feature of Office Excel 2016 (Microsoft Corporation©, Redmond, WA, USA). The corresponding results (PEEK/titanium) were placed in consecutively numbered envelopes. These were opened by the operating surgeon immediately before the surgical procedure.

The implants, made of carbon fiber-reinforced (CFR = carbon fiber reinforced) polyetheretherketone (PEEK), are characterized by a stiffness that is adapted to human bones. The CFR-PEEK plate consists of 55–60% carbon fiber. The random arrangement of these fibers within the plate contributes to the bone-adapted biomechanical properties described in the introduction. The remaining 40–45% of the plate is made of polyetheretherketone.

On the one hand, the new material allows interfragmentary micro-movements, which are intended to promote faster callus formations. On the other hand, the material is transparent to X-rays, which might lower the risk of primary unnoticed screw perforations. Furthermore, the rate of secondary screw perforations could also be reduced by adapting the stiffness of the implant to the bone. Similar to the PHILOS plate, the CFR-PEEK plate is adapted to the anatomical shape of the proximal humerus. There are seven screw holes in the proximal part of the plate so that screws can be inserted polyaxially. There are two types of plates available, which differ in the length of the part used to stabilize the shaft fragment. With the shorter version, three screws can be inserted into the shaft whereas the longer version allows stabilization to shaft with up to five screws. Titanium screws are used for the CFR-PEEK plate system, which has a core diameter of 4.0 mm in the head area and a core diameter of 3.5 mm in the shaft area. The CFR-PEEK plate offers the surgeon the opportunity to vary the insertion of the angle-stable screws with an angular deviation of up to 12°. This allows the screws to be placed individually to suit the anatomical conditions. Comparable with other modern plating systems, holes are provided for the attachment of suture cerclages for additional fragment stabilization.

The surgical procedure and osteosynthesis technique did not differ when using the CFR-PEEK and the PHILOS plate. The patients were positioned in beach chair position under full anesthesia. The anterolateral approach according to McKenzie was performed, characterized by the skin incision starting at the coracoid parallel to the axillary fold and the subsequent blunt cutting in the direction of the fibers of the deltoid muscle.

Under visualization of the fracture, the greater and lesser tuberosities were first addressed using non-absorbable sutures (FibreWire, Arthrex, Naples, FL, USA). The fracture fragments were then anatomically reduced and temporarily fixed using K-wires. The plate osteosynthesis was attached five to eight millimeters distal to the tip of the greater tuberosity and directly lateral to the bicipital groove. The plate was always fixed to the humeral shaft using a cortical screw and two angle-stable screws. Only angle-stable screws were used in the area of the humeral head. However, the number of these was variable and selected individually depending on the fracture. Furthermore, the FibreWires were fixed to the plate. The anatomical reduction and the correct implant position were checked intraoperatively using an image intensifier. All patients received a Gilchrist bandage for 7–10 days, which had to be worn permanently. In the following two weeks, the range of motion of the shoulder joint was increased to a maximum of 60° anteversion and abduction. External rotation movements and retroversion were not allowed. Anteversion and abduction were then limited to 90° and external rotation and retroversion to 20° for another two weeks. After this time, the glenohumeral joint was released to its full range of motion with a limited weight-bearing of the operated arm of 15 kg for 6 weeks postoperatively.

Bilateral or previous humerus fractures, head-split fractures, patients with cuff arthropathies, nerve or vascular injuries, thrombophilia, severe cardiac or pulmonary disease, and alcohol or drug abuse were all exclusion criteria. The results for bony consolidation and early postoperative outcomes have already been published by Ziegler et al. [25].

In addition to assessing the functional outcome, demographic data such as age, gender, body mass index, fracture type, and co-morbidities were also recorded. Functional outcome was determined using the DASH score, Simple Shoulder Test, and the Oxford Shoulder Score at 6 weeks, 12 weeks, 6 months, and 12 months post-operative. The scores described are accepted analysis methods that are used frequently in the literature.

Sample size planning was based on an assumed mean difference between the DASH scores of 5 points with a range of ±18 points. Based on a desired power of 80%, a sample size of *n* = 30 patients per group (30 CFR-PEEK and 30 titanium) was calculated. For planning, the independent two-sample *t*-test was used.

The 2 study groups were treated with 2 different plates: The locking CFR-PEEK plate (PEEKPower Humeral Fracture Plate, Arthrex, Naples, FL, USA) and a locking titanium plate (Depuy Synthes, Proximal Humerus Internal Locking System—PHILOS, West Chester, PA, USA). More detailed information on the surgical procedure and post-op follow-up can be found in the previously published paper from the working group [25]. 

### Statistics

All obtained data were documented descriptively. Continuous variables were reported as means ± standard deviation. For dichotomous/categorical variables, frequencies and percentage shares, respectively, were reported. For the comparison of baseline characteristics, a two-sided significance level was used.

The statistical analysis was performed using SPSS (Version 24, SPSS Inc., Chicago, IL, USA). The independent two-sample *t*-test was used to analyze potential differences between the two groups with respect to the primary endpoint. The postoperative head–shaft angle measurements were evaluated using repeated measures analysis of variance. Potential preoperative differences between the two groups were calculated using the independent samples *t*-test (age, BMI), Fisher‘s exact test (comorbidities), or the chi-squared test (sex, fracture type, ASA classification). Values of *p* < 0.05 were regarded as significant.

All patients for whom data from at least one follow-up time point were available were included in the analysis. Missing data were not replaced. As a sensitivity analysis with respect to the primary endpoint (DASH), the independent two-sample *t*-test was used with the method of multiple imputations (*n* = 100), based on all randomized patients. The *t*-test was applied to 2 independent samples in order to identify possible significant differences in the functional outcome. The distribution of the independent samples was a result of the respective plate treatment type (CFR-PEEK; titanium). The significance level was set at *p* < 0.05.

## 3. Results

A total of 54 patients were included 1 year postoperatively in this prospective, randomized study. The average age was 62.65 ± 11.34 years (Table 1). The distribution of fracture severity based on the Neer classification showed a comparable number of II part fractures in both groups and a higher number of III part fractures with a simultaneously lower number of IV part fractures in the CFR-PEEK group compared with the titanium group (Table 1). Of the 54 patients, 29 (53.57%) were treated with a CRF-PEEK plate and 25 (46.43%) with a titanium plate.

A total of 22 patients were lost to follow-up after 1 year. Two patients were already excluded intraoperatively due to a head-split component of the fracture. Two further patients had a second accident after surgery and required revision surgery. Eighteen patients declined further study participation without any reason. At the follow-up appointments 6 and 12 weeks post-op, the functional outcome of 63 patients (*n* = 32–50.80% CFR-PEEK; *n* = 31–49.20% titanium; follow-up rate 82.89%) could be analyzed. One-year outcomes could be obtained for 54 patients (*n* = 29–53.70% CFR-PEEK; *n* = 25–46.29% titanium, follow-up rate 71.05%). The two groups did not differ significantly in terms of distribution of age, BMI, handedness, or secondary disease, as defined by the ASA classification.

### Functional Outcome

**Figure 1:**
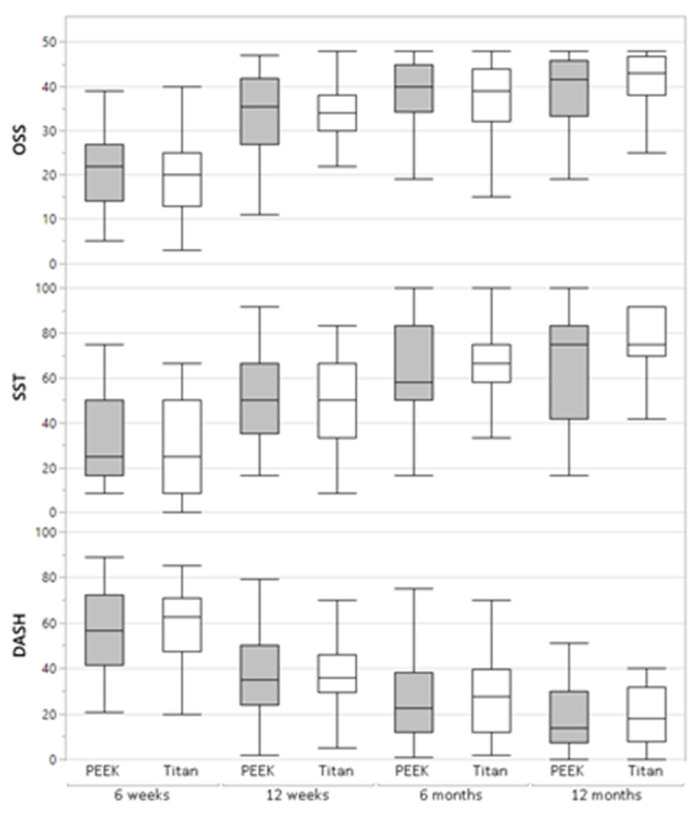
Differences in functional outcome regarding treatment with the different plates.

One year post-op, all patients demonstrated a significantly improved functional outcome compared with the previous follow-up examination at 6 weeks, 12 weeks, and 6 months post-op (Table 2 and Table 3). The CFR-PEEK group reached 18.6 ± 14.7 points in the DASH Score, and the titanium group 23.9 ± 22.0 points. Similar results were also seen in the Simple Shoulder Test (71.5 ± 18.2 CFR-PEEK; 71.3 ± 22.8 titanium) and the Oxford Shoulder Score (38.4 ± 12.2 CFR-PEEK; 39.3 ± 8.6 titanium) (Table 2, Figure 1). No significant differences could be identified regarding treatment with the different plates.

## 4. Discussion

This prospective, randomized study was conducted in order to evaluate whether the use of CFR-PEEK results in a change in the functional postoperative outcome compared to a titanium plate for the surgical treatment of proximal humerus fractures. Neither investigators nor patients could be blinded during the follow-up period. The study design did not allow for conclusions on the equivalence of the two interventions. That would have required a non-inferiority study design.

Since CFR-PEEK is radiotranslucent there is no superimposition of portions of the proximal humerus during intraoperative and postoperative imaging. Bony consolidation of the respective fracture was confirmed in all the patients included in the study within the scope of postoperative follow-up care. Within the scope of the functional outcome assessed using the DASH score, Oxford Shoulder Score, and the Simple Shoulder Test, no significant differences were detected between the implant materials CFR-PEEK and titanium for the treatment of proximal humerus fractures.

Various implants are available for the osteosynthetic treatment of proximal humerus fractures. Open reduction and stabilization with a locking plate are often the treatment of choice for multi-fragmented or displaced fractures of the proximal humerus [13,26]. The plates used for these fractures have different material properties. Plates made of steel, titanium, and CFR-PEEK are used most frequently. The cited advantages of CFR-PEEK over titanium or steel are radiolucency and no risk of screw–plate cold welding as is the case with titanium screw and plate combinations, i.e., the joining of two metallic workpieces of the same material at room temperature. In addition, the increased biomechanical elasticity of the CFR-PEEK plate may reduce stress-shielding at the plate–bone junction and offer a positive effect on bony consolidation through micro-motion. 

An increase in the incidence of proximal humerus fractures has been observed in recent years. As demographic change and life expectancy continue to increase, the optimal treatment of proximal humerus fractures will become increasingly important. Therefore, treatment of proximal humerus fractures remains subject to constant change. Attempts are being made to reduce the high complication rates associated with the use of new implant materials. Complications such as primary screw perforation, misplacement of the plate, or loss of reduction due to lack of medical support can be avoided by optimizing the surgical technique. Thanks to the radiolucent nature of the CFR-PEEK plate, all screws used can be visualized without superimposition. Loss of reduction due to the high stiffness of titanium and steel locking plates may lead to failure at the bone–screw interface, particularly in osteoporotic bone. This occurrence can be reduced by the increased elasticity of the CFR-PEEK plate. Lill et al. examined the initial stiffness of various implants for the treatment of proximal humerus fractures [27]. They discovered that implants that are less stiff and more elastic seem to reduce peak stress at the bone–implant interface, making them suitable for fracture fixation in osteoporotic bone. Schliemann et al. documented less frequent secondary varus dislocations following treatment of a proximal humerus fracture with CFR-PEEK plate compared with an independent group which was surgically treated with titanium implants [22]. No statistically significant differences in terms of the functional postoperative outcome were found in our study population. This was also confirmed by other authors in further studies [21,28]. 

Studies on the long-term outcomes using CFR-PEEK plates in osteoporotic bone should be the subject of further research. 

## 5. Conclusions

No significant differences could be detected in terms of functional outcome between CFR-PEEK plates and titanium implants 1 year after surgery. Studies on the long-term outcomes using CFR-PEEK plates in osteoporotic bone should be the subject of further research.

## Figures and Tables

**Table 1:** Demographic data age (**a**), gender (**b**), fracture classification (**c**).

**(a)**			
	**Average**	**Standard Deviation**	**Median**
**Age Overall Collective (1-Year-Follow-Up)**	62.65	11.34	61
**Age Titanium Collective (1-Year-Follow-Up)**	62.80	9.79	62
**Age PEEK Collective (1-Year Follow-Up)**	62.52	12.53	61
**(b)**			
**Gender**	**PEEK**		**Titanium**
**Female**	24 (82.8%)		21 (84.0%)
**Male**	5 (17.2%)		4 (16.0%)
**(c)**			
**Neer-Classification**	**PEEK**		**Titanium**
**2-Part**	6 (20.7%)		3 (12.0%)
**3-Part**	19 (65.5%)		13 (52.0%)
**4-Part**	4 (13.8%)		9 (36.0%)

**Table 2:** Differences in functional outcome at follow-up dates. ns: non-significant.

		PEEK	Titan
Questionnaire	Time Point	*p*-Value	*p*-Value
OSS	6 w–12 m	<0.0001	<0.0001
	6 w–6 m	<0.0001	<0.0001
	6 w–12 w	<0.0001	<0.0001
	12 w–12 m	ns	0.0201
	12 w–6 m	ns	0.0358
SST	6 w–12 m	<0.0001	<0.0001
	6 w–6 m	<0.0001	<0.0001
	6 w–12 w	<0.0001	0.0001
	12 w–12 m	0.0185	0.0014
	12 w–6 m	ns	0.0363
DASH	6 w–12 m	<0.0001	<0.0001
	6 w–6 m	<0.0001	<0.0001
	6 w–12 w	0.0006	<0.0001
	12 w–12 m	0.0015	0.0264

**Table 3:** Differences in functional outcome at follow-up dates.

		PEEK		Titan	
Questionnaire	Time Point	Mean ± SD	Median (Min–Max)	Mean ± SD	Median (Min–Max)
OSS	6 weeks	20.3 ± 9.8	20.5 (3.0–40.0)	20.4 ± 8.5	21.5 (3.0–39.0)
	12 weeks	33.8 ± 10.0	35.5 (11.0–47.0)	33.3 ± 6.5	34.0 (17.0–48.0)
	6 months	37.7 ± 8.8	40 (15.0–48.0)	38.6 ± 6.8	39.0 (19.0–48.0)
	12 months	38.4 ± 12.2	43 (22–48.0)	39.3 ± 8.6	42 (19.0–48.0)
SST	6 weeks	30.0 ± 20.8	29.2 (0.0–75.0)	29.4 ± 18.9	25.0 (0.0–75.0)
	12 weeks	54.9 ± 24.8	54.2 (8.3–91.7)	51.5 ± 16.5	50.0 (16.7–83.3)
	6 months	62.5 ± 22.3	61.8 (18.2–100)	65.0 ± 20.1	58.3 (16.7–100.)
	12 months	71.5 ± 18.2	75 (33.3–100)	71.3 ± 22.8	75 (16.7–100.)
DASH	6 weeks	56.5 ± 19.3	56.9 (20.7–88.9)	59.8 ± 15.6	62.5 (19.8–85.3)
	12 weeks	38.4 ± 21.4	35.1 (1.7–79.3)	37.7 ± 16.2	35.8 (5.2–73.3)
	6 months	27.5 ± 20.5	22.4 (1.0–81.5)	28.5 ± 17.9	27.6 (1.7–69.8)
	12 months	18.6 ± 14.7	13.8 (0.0–50.9)	23.9 ± 22.0	17.9 (0.0–78.4)

## Data Availability

Data have not been deposited into a public repository but are available upon request.

## Abbreviations

ASA	American Society of Anesthesiologists
BMI	Body Mass Index
DASH-Score	Disabilities of the Arm, Shoulder and Hand-Score
CFR-PEEK	carbon fiber reinforced polyetheretherketone
PHILOS	proximal humerus internal locking system

## References

[1] Baron J.A., Karagas M., Barrett J., Kniffin W., Malenka D., Mayor M., Keller R.B. (1996). Basic epidemiology of fractures of the upper and lower limb among Americans over 65 years of age. Epidemiology.

[2] Bell J.E., Leung B.C., Spratt K.F., Koval K.J., Weinstein J.D., Goodman D.C., Tosteson A.N. (2011). Trends and variation in incidence, surgical treatment, and repeat surgery of proximal humeral fractures in the elderly. J. Bone Jt. Surg..

[3] Court-Brown C.M., McQueen M.M. (2016). Global Forum: Fractures in the Elderly. J. Bone Jt. Surg..

[4] Passaretti D., Candela V., Sessa P., Gumina S. (2017). Epidemiology of proximal humeral fractures: A detailed survey of 711 patients in a metropolitan area. J. Shoulder Elb. Surg..

[5] Gupta A.K., Harris J.D., Erickson B.J., Abrams G.D., Bruce B., McCormick F., Nicholson G.P., Romeo A.A. (2015). Surgical management of complex proximal humerus fractures-a systematic review of 92 studies including 4500 patients. J. Orthop. Trauma.

[6] Konigshausen M., Kubler L., Godry H., Citak M., Schildhauer T.A., Seybold D. (2012). Clinical outcome and complications using a polyaxial locking plate in the treatment of displaced proximal humerus fractures. A reliable system?. Injury.

[7] Schliemann B., Siemoneit J., Theisen C., Kosters C., Weimann A., Raschke M.J. (2012). Complex fractures of the proximal humerus in the elderly—Outcome and complications after locking plate fixation. Musculoskelet. Surg..

[8] Schliemann B., Wähnert D., Theisen C., Herbort M., Kösters C., Raschke M.J., Weimann A. (2015). How to enhance the stability of locking plate fixation of proximal humerus fractures? An overview of current biomechanical and clinical data. Injury.

[9] Acklin Y.P., Stoffel K., Sommer C. (2013). A prospective analysis of the functional and radiological outcomes of minimally invasive plating in proximal humerus fractures. Injury.

[10] Brunner F., Sommer C., Bahrs C., Heuwinkel R., Hafner C., Rillmann P., Kohut G., Ekelund A., Muller M., Audigé L. (2009). Open reduction and internal fixation of proximal humerus fractures using a proximal humeral locked plate: A prospective multicenter analysis. J. Orthop. Trauma.

[11] Falez F., Papalia M., Greco A., Teti A., Favetti F., Panegrossi G., Casella F., Necozione S. (2016). Minimally invasive plate osteosynthesis in proximal humeral fractures: One-year results of a prospective multicenter study. Int. Orthop..

[12] Handschin A.E., Cardell M., Contaldo C., Trentz O., Wanner G.A. (2008). Functional results of angular-stable plate fixation in displaced proximal humeral fractures. Injury.

[13] Tepass A., Blumenstock G., Weise K., Rolauffs B., Bahrs C. (2013). Current strategies for the treatment of proximal humeral fractures: An analysis of a survey carried out at 348 hospitals in Germany, Austria, and Switzerland. J. Shoulder Elb. Surg..

[14] Konrad G., Bayer J., Hepp P., Voigt C., Oestern H., Kääb M., Luo C., Plecko M., Wendt K., Köstler W. (2010). Open reduction and internal fixation of proximal humeral fractures with use of the locking proximal humerus plate. Surgical technique. J. Bone Jt. Surg..

[15] Owsley K.C., Gorczyca J.T. (2008). Fracture displacement and screw cutout after open reduction and locked plate fixation of proximal humeral fractures [corrected]. J. Bone Jt. Surg..

[16] Spross C., Platz A., Erschbamer M., Lattmann T., Dietrich M. (2012). Surgical treatment of Neer Group VI proximal humeral fractures: Retrospective comparison of PHILOS(R) and hemiarthroplasty. Clin. Orthop. Relat. Res..

[17] Fu T., Xia C., Li Z., Wu H. (2014). Surgical versus conservative treatment for displaced proximal humeral fractures in elderly patients: A meta-analysis. Int. J. Clin. Exp. Med..

[18] Okike K., Lee O.C., Makanji H., Morgan J.H., Harris M.B., Vrahas M.S. (2015). Comparison of locked plate fixation and nonoperative management for displaced proximal humerus fractures in elderly patients. Am. J. Orthop..

[19] Olerud P., Ahrengart L., Ponzer S., Saving J., Tidermark J. (2011). Internal fixation versus nonoperative treatment of displaced 3-part proximal humeral fractures in elderly patients: A randomized controlled trial. J. Shoulder Elb. Surg..

[20] Padolino A., Porcellini G., Guollo B., Fabbri E., Kiran Kumar G.N., Paladini P., Merolla G. (2018). Comparison of CFR-PEEK and conventional titanium locking plates for proximal humeral fractures: A retrospective controlled study of patient outcomes. Musculoskelet. Surg..

[21] Rotini R., Cavaciocchi M., Fabbri D., Bettelli G., Catani F., Campochiaro G., Fontana M., Colozza A., De Biase C.F., Ziveri G. (2015). Proximal humeral fracture fixation: Multicenter study with carbon fiber peek plate. Musculoskelet. Surg..

[22] Schliemann B., Hartensuer R., Koch T., Theisen C., Raschke M.J., Kösters C., Weimann A. (2015). Treatment of proximal humerus fractures with a CFR-PEEK plate: 2-year results of a prospective study and comparison to fixation with a conventional locking plate. J. Shoulder Elb. Surg..

[23] Schliemann B., Seifert R., Theisen C., Gehweiler D., Wähnert D., Schulze M., Raschke M.J., Weimann A. (2017). PEEK versus titanium locking plates for proximal humerus fracture fixation: A comparative biomechanical study in two- and three-part fractures. Arch. Orthop. Trauma Surg..

[24] Katthagen J.C., Schwarze M., Warnhoff M., Voigt C., Hurschler C., Lill H. (2016). Influence of plate material and screw design on stiffness and ultimate load of locked plating in osteoporotic proximal humeral fractures. Injury.

[25] Ziegler P., Maier S., Stockle U., Guhring M., Stuby F.M. (2019). The Treatment of Proximal Humerus Fracture Using Internal Fixation with Fixed-angle Plates. Dtsch. Arztebl. Int..

[26] Arbeitsgemeinschaft der Wissenschaftlichen Medizinischen Fachgesellschaften (2017). S1 Leitlinie: Oberarmkopffraktur.

[27] Lill H., Hepp P., Korner J., Kassi J.P., Verheyden A.P., Josten C., Duda G.N. (2003). Proximal humeral fractures: How stiff should an implant be? A comparative mechanical study with new implants in human specimens. Arch. Orthop. Trauma Surg..

[28] Katthagen J.C., Ellwein A., Lutz O., Voigt C., Lill H. (2017). Outcomes of proximal humeral fracture fixation with locked CFR-PEEK plating. Eur. J. Orthop. Surg. Traumatol..

